# Reasons for living and depressive symptomatology in young adults with and without suicide attempts: a moderated mediation approach

**DOI:** 10.3389/fpsyt.2024.1443894

**Published:** 2025-01-23

**Authors:** Marta Brás, Cátia Martins, Cristina Nunes, Saul Neves Jesus, Ana Rita Madeira, Cláudia Carmo

**Affiliations:** ^1^ Psychology and Education Sciences Department, University of Algarve, Faro, Portugal; ^2^ Psychology Research Centre (CIP), Lisboa, Portugal; ^3^ University Research Center in Psychology (CUIP), Faro, Portugal; ^4^ Research Centre for Tourism, Sustainability and Well-being (CinTurs), Faro, Portugal

**Keywords:** suicide, depressive symptoms, reasons for living, hopelessness, young adults

## Abstract

**Introduction:**

Suicide is a public health problem worldwide, being the fourth leading cause of death in young adult population (15-29 years). Therefore, it is important to identify its risk and protective factors, and how they interact to develop more effective interventions. The present study aims to analyze the relation between depressive symptoms, hopelessness (risk factors) with reasons for living (protective factors) and suicidal ideation in young adults with and without previous suicide attempts.

**Methods:**

A sample of 845 Portuguese young adults answered an online form which assessed suicidal ideation, hopelessness, depressive symptoms, and reasons for living. Mean comparisons, correlations, and moderated mediation analysis were computed.

**Results:**

Individuals with a history of suicide attempt showed higher mean levels in risk factors, compared to individuals without suicide attempt. When no history of suicide attempt was present, ideation was positively and significantly correlated to depression and hopelessness, and negatively correlated to the reasons for living. Higher levels of depressive symptomatology predict lower levels of reasons for living. Mediation and moderation relations were assessed in a model and the history of suicide attempts showed a relevant role.

**Conclusions:**

The evidence found in this study reinforce that reasons for living may play a preponderant role in suicidal ideation, that is as a negative mediator and therefore it can act as a protective factor.

## 1 Introduction

Suicide is a public health problem worldwide, being the third leading cause of death in young adult population (aged 15-29 years) (1). In Portugal it is also one of the main causes of death among young adults, with a mortality rate of 9 per 100,000 inhabitants in 2020 (2).

Bagge et al. (3) report that approximately 20% of college students have considered committing suicide and more than 7% have attempted suicide. These rates are most likely underestimated because suicides are reported as other causes of death, such as traffic accidents (4–6).

Regarding the pandemic situation, several researchers identified a rise in loneliness and isolation in these age groups, increasing the likelihood of a scenario of suicidal ideation or suicidal behaviors in which to mobilize the necessary help can be difficult (7, 8). Depressive and anxious symptomatology have also increased with the passing of the pandemic, presenting a higher incidence in young adults, when compared to other age groups, which also can enhance the risk of suicide (7).

Suicide is a multifaceted and complex phenomenon that corresponds, most of the time, to the end of a process that range in a continuum from suicidal ideation to the completed suicide (9, 10). The General Health Coordination [GHC; (9)], in the National Suicide Prevention Plan, defines suicidal ideation as thoughts that aim to end one’s life, which may or may not be proceeded by a suicide attempt. Suicide attempt is understood as any act carried out by the self with the purpose of death, but which, for various reasons, results in the failure of its execution. In this process, self-injurious behaviors may coexist, which consist of behavioral acts against oneself, but without the intention to cause death (9).

Initiating the suicidal process seems to represent an increased risk for future suicide, especially if suicide attempts occur. It is estimated that 15% of individuals who commit one or more suicide attempts will perform the consummated suicide (11), and this being considered the main predictor (11, 12).

Given the importance of preventing these behaviors, research has been devoted to explore risk factors associated with suicide and more recently with protective factors; however, the need for robustness in this research topic is still imperative (13–15). Franklin et al. (16) in a meta-analysis conducted about risk factors for suicidal behaviors and thoughts reported a discrepancy between studies on risk factors, about more than three thousand studies, and studies on protective factors, less than five hundred studies. In addition, the observed decrease falls short of what is expected by 2030 by the World Health Organization, as a plan of action and prevention in reducing suicide (1).

Given the discrepancy between the number of studies on risk and protective factors, the focus is currently on protective factors and their possible mediating roles. Understanding the risk factors that increase the risk for suicidal ideation, suicide attempt, and completed suicide may not be sufficient to minimize the risk of these behaviors (10, 17). There is consistent empirical evidence on the role of psychological risk factors, such as depressive symptomatology and hopelessness (18, 19), but the role of protective factors, such as reasons for living is not fully clarified, especially regarding the interaction with other risk factors. Reasons for living (RFL) consist of the beliefs that an individual holds about his or her own life and expectations that seem to mitigate suicidal ideation (13, 20).

Individuals who present hopelessness regarding the future and/or have depressive symptomatology may have difficulty in identifying, enumerating, or connecting their RFL, which may result in suicidal ideation and/or an increased likelihood of a suicide attempt (3, 21). Furthermore, individuals with suicidal ideation reveal low levels of RFL compared to those without such ideation, and the ones with suicidal ideation generally lack cognitive beliefs that support the idea that they have reasons not to consummate suicide (22). The presence of more severe depressive symptoms and hopelessness, marked by high levels of mental pain, has been identified as a risk factor for suicide (4).

Ren et al. (23) found that RFL in adolescents is negatively related to low self-esteem, perceived inability to escape from aversive situations, and suicidal ideation. The association between perceived inability to escape from aversive situations and suicidal ideation is moderated by RFL. This study thus suggests that RFL may weaken the relationship between risk factors for suicide and suicidal ideation.

The prevalence and increasing rates of suicidal behavior and ideation in college students remains significant and leads to the emergence for mental health professionals. Given the negative impact and often unalterable consequences that suicide can cause, it is essential to understand the reasons why young-adults consider life worth living (13).

Wang and colleagues (25) showed that severe depression has a mediating effect on the relationship between hopelessness and suicidal ideation, that is, hopelessness does not seem to be a direct cause of suicidal ideation, but if individuals with hopelessness are in depressive states, they are more likely to have more thoughts related to suicide. Hope provides a perception of control over one’s life, making it easier to find alternative solutions when faced with problematic situations. Additionally, because they have high levels of hopelessness, individuals at risk of suicide usually have difficulty solving problems (24). They also compared the levels of hopelessness in individuals with and without suicidal ideation and concluded that the former showed higher levels of hopelessness (25).

However, divergent results on the role of RFL in suicide emerge in the literature (17). In fact, several factors have been identified that, when interacting with each other, they can contribute to the development of suicidal behaviors. A history of prior suicide attempt, especially in the past 6 months, is such a factor, that it is considered more important than any psychiatric disorder (11, 12). Even so, Klonsky and May (10) refer the existence of contradictory results, with differences between clinical and non-clinical populations, but without statistically significant differences in RFL between individuals with suicidal ideation alone and with depressive symptomatology.

Since suicide attempts are considered the best predictor of future suicidal acts, it is important to clarify whether they have a differential effect on the relationship between other risk and protective factors for suicidal ideation. That is, if the relationship between depressive symptoms, reasons for living and suicidal ideation varies according to the existence of previous suicide attempts.

**Figure 1:**
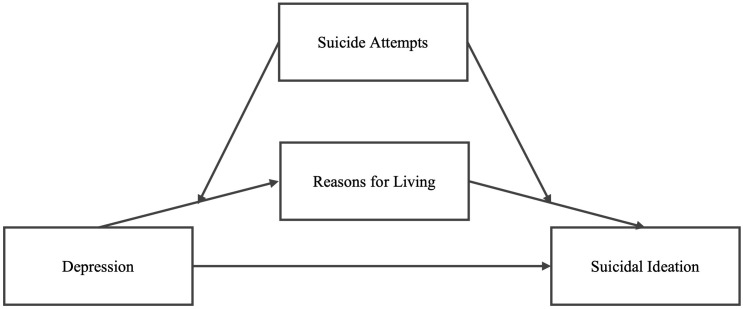
Suicidal Ideation conceptual model to be tested.

The present study aims to analyze the relation between depressive symptoms, hopelessness (risk factors for suicide) with reasons for living (protective factors) and suicidal ideation in young adults with and without previous suicide attempts (**Figure 1**).

## 2 Methods

### 2.1 Participants

The study was conducted with a national sample of 845 young adults, from different regions of Portugal and islands, where 189 were males (22.4%) and 656 females (77.6%). 33 participants (3.9%) had previously attempted suicide and 812 participants had not (96.1%). Age ranked from 18 to 30 years old (*M* = 21.79; *DP* = 2.87).

### 2.2 Instruments

#### 2.2.1 Sociodemographic questionnaire

The sociodemographic and clinical information questionnaire was developed to gathered information regarding (a) personal information (age, gender, education level); (b) history of psychopathology; (c) history of suicidal behavior including previous suicide attempts; and (d) history of suicide attempts in family members or close ones.

#### 2.2.2 Suicidal Ideation Questionnaire (SIQ)

The SIQ (26, 27) main objective is to assess the severity of suicidal thoughts in adolescents. It is composed of 30 items (e.g., “I thought about killing myself”), and each one can be punctuated from 0 (Never had this thought) to 6 (Almost every day). The final score can range from 0 to 180 points; and the higher the score, the more frequent are the suicidal thoughts. The Portuguese version of this questionnaire has good psychometrics values (*α* = .96) (26).

#### 2.2.3 Beck Hopelessness Scale (BHS)

BHS (28, 29) is a 20 items-scale that assesses hopelessness or negative attitudes about future related events answered with single choice option, true or false (e.g., “I look forward to the future with hope and enthusiasm”; “My future seems dark to me”). The total score ranges from 0 to 20, and higher scores are associated with more negative attitudes toward the future (28). The Portuguese version was used, and the original study reveal good internal consistency (*KR_20* = .90) (29).

#### 2.2.4 Beck Depression Inventory – II (BDI-II)

BDI-II (30, 31) is a 21 items-inventory that assesses the severity of depressive symptoms. Each item is rated on a 4-point scale ranging from 0 to 3, with a maximum total score of 63 points (e.g., “I do not feel sad”). The higher the total score is, the severe the symptoms are (30). The Portuguese version of BDI-II has good internal reliability (*α* = .91), identical factorial structure to the original study and adequate convergent validity (31).

#### 2.2.5 Reasons for Living Inventory for Young Adults (RFL-YA-II)

The inventory RFL-YA-II (6, 13, 32) has 28 items organized in four subscales: (1) Faith-Related Support (FRS; e.g., “I believe that I can find a solution to most problems because of my faith”); (2) Family Sources of Support (FSS; e.g., “I have a close relationship with my family”); (3) Perceived Personal Strength (PPS; e.g., “I feel satisfied with myself”); and (4) Peer Acceptance and Support (PAS; e.g., “believe that I can count on my friends when I have a problem”). Each item is answered from 1 (Not at all important) to 6 (Extremely important). The Cronbach’s alpha of the Portuguese version indicated values similar with the original study, suggesting good consistency (between.93 and.98) (6, 13, 32).

### 2.3 Data collection procedure

The data collection protocol was disseminated through an online form by several higher education institutions in Portugal. Participation was anonymous, confidential, and voluntary, without any counterpart and preceded by informed consent. To avoid more than one answer by the same participants to the survey, emails were collected. According to WHO information (1) regarding the age of young people at which the highest number of suicides occurs, inclusion criteria were age from 18 to 30 years and Portuguese nationality. Scientific and ethical approvals were sought from the Faculty of Human and Social Sciences of the University of Algarve.

### 2.4 Data analyses procedure

Data were analyzed using the Statistical Package for the Social Sciences (SPSS) software, version 28.0 for Windows. Descriptive statistics (i.e., frequency, percentage, mean and standard deviation) were performed for the sociodemographic variables. Student’s t test for independent samples was used to compare individuals with and without a history of suicide attempt on the variables of interest: suicidal ideation, depressive symptoms, hopelessness, and reasons for living. After the confirming the assumptions of normality and homogeneity, results were interpreted regarding 5% significance level and Cohen’s *d* was used to analyze the magnitude of the differences (0.25 indicating a low effect, 0.50 a moderate effect, and 0.80 a high effect). Pearson’s correlation coefficient was computed to analyze the relationship between the variables of interest, and significance (5%), magnitude and sign were explored (33).

Lastly, moderated mediation analysis was carried out with model 58 of Hayes PROCESS macro for SPSS (34). In this model, suicidal ideation (total score) was entered as the outcome variable, depressive symptomatology as independent variable, and the reasons for living (total score) as mediator. History of suicidal attempt was included as the moderator on the dependent variables’ reasons for living and suicidal ideation. See **Figure 1** for a representation of the conceptual and statistic model.

## 3 Results

The mean values of suicidal ideation, depressive symptomatology, hopelessness, and reasons for living were compared between individuals with and without a history of suicide attempt (**Table 1**).

**Table 1:** Means, Standard Deviations, Cohen’s d and Student’s t-test of the variables according to the history of suicide attempts.

Variables	Individuals with suicide attempt (n = 33)		Individuals without suicide attempt (n = 812)		t	p	Cohen’s d
	*M*	*SD*	*M*	*SD*			
1.Ideation	50.78	41.57	14.71	19.90	-4.96	<.001	-1.71
2.Depression	24.27	16.30	10.20	9.86	-4.92	<.001	-1.38
3.Hopelessness	7.81	4.91	4.43	3.45	-3.93	<.001	-.97
4.RFL-YA-Total	94.39	32.14	124.56	21.67	5,34	<.001	1.34
FRS	15.18	10.71	20.48	10.85	2.75	.24	.49
PAS	28.64	9.24	34.22	6.10	3.44	<.001	.90
FSS	25.67	11.50	35.25	6.59	4.76	<.001	1.40
PPS	24.91	10.84	34.61	6.29	5.11	<.001	1.49

*N* = 845; *M* = Mean; *SD* = Standard Deviation; *t* = Student’s *t* test for independent samples; RFL -YA-Total = Reasons for Living-Young Adults-Total; FRS = Faith-Related Support – subscale of RFL-YA-II; PAS = Peer Acceptance Support - subscale of RFL-YA-II; FSS = Family Sources of Support - subscale of RFL-YA-II; PPS = Perceived Personal Strength – subscale of RFL-YA-II.

As shown in **Table 1**, results reveal significative differences in all dimensions, except in faith-related support (*t* = 2.75, *p* = .240, *d* = .49), but with a moderate effect. So, individuals with a history of suicide attempt show higher mean levels of suicidal ideation (*M*_With_ = 50.78, *SD*_With_ = 41.57; *M*_Without_ = 14.71, *SD*_Without_ = 19.90), depressive symptoms (*M*_With_ = 24.27, *SD*_With_ = 16.30; *M*_Without_ = 10.20, *SD*_Without_ = 9.86), hopelessness (*M*_With_ = 7.81, *SD*_With_ = 4.91; *M*_Without_ = 4.43, *SD*_Without_ = 3.45), and reasons for living (*M*_With_ = 94.39, *SD*_With_ = 32.14; *M*_Without_ = 124.56, *SD*_Without_ = 21.67) compared to individuals without suicide attempt, in the risk factors. Thus, those who have history of suicide attempt may be more susceptible to suicidal thoughts/behaviors, depressive symptoms, and hopelessness, which leads to a higher risk of suicide. In relation to reasons for living, individuals with history of suicide attempt present a lower mean value than individuals without history of suicide attempt. The group of people with a history of suicide attempt scored higher on the PAS (*M*_With_ = 28.64, *SD*_With_ = 9.24; *M*_Without_ = 34.22, *SD*_Without_ = 6.10) and FSS (*M*_With_ = 25.67, *SD*_With_ = 11.50) subscales of the RFL-YA-II, while individuals without a history of suicide attempt had higher levels on the FSS (*M*_Without_ = 35.25, *SD*_Without_ = 6.59) and PPS subscales (*M*_With_ = 24.91, *SD*_With_ = 10.84; *M*_Without_ = 34.61, *SD*_Without_ = 6.29).

As for the effect size, it is moderate only in the FRS (*d* = -.97), and the rest of the analyzed dimensions, its score is very large (*d* = .90 - 1.71).

For the individuals without history of suicide attempt (**Table 2**, left triangle), ideation was highly and positively correlated to depression (*r*_without_ = .71, *p* <.001) and moderately to hopelessness (*r*_without_ = .57, *p* <.001), and low and negative correlated to the total RFL (*r*_without_ = -.33, *p* <.001), and respective sub-scales (*r_WhithoutFRS_* = -.16, *p* <.001; *r_FRS_* = -.16, *p* <.001). Depression was positively moderately correlated to ideation (*r*_without_ = .64, *p* <.001) and hopelessness (*r*_without_ = .67, *p* <.001), and negatively with RFL (*r*_without_ = -.36, *p* <.001). Hopelessness displays low negative correlations to the total score of RFL (*r*_without_ = -.35, *p* <.001), and its sub-scales (*Range r*_without_ = -.21 - -.36, *p* <.001).

**Table 2:** Intercorrelations of study variables separated by history of suicidal attempt (with and without).

With \ Without	1	2	3	4	5	6	7	8
1.Ideation	–	.64**	.51**	-.39*	-.27	-.20	-.20	-.49**
2.Depression	.71**	–	.89**	-.34	-.07	-.16	-.17	-.60**
3.Hopelessness	.57**	.67**	–	-.40*	-.07	-.19	-.29	-.65**
4.RFL-YA-Total	-.33**	-.36**	-.35**	–	.59**	.82**	.58**	.79**
5.FRS	-.16**	-.14**	-.21**	.72**	–	.27	.33	.17
6.PAS	-.25**	-.29**	-.26**	.70**	.20**	–	.64**	.62**
7.FSS	-.29**	-.25**	-.24**	.76**	.33**	.50**	–	.59**
8.PPS	-.34**	-.44**	-.36**	.72**	.23**	.58**	.50**	–

N = 845; Ideation =Suicidal Ideation Questionnaire; Depression = Beck Depression Inventory; Hopelessness = Beck Hopelessness Scale; RFL-YA-II = Reasons for Living Inventory total score; FRS RFL-YA = Faith-Related Support; PAS RFL-YA-II = Peer Acceptance and Support; FSS RFL-YA-II = Family Sources of Support; PPS RFL-YA-II = Perceived Personal Strength. Correlations for participants with suicide attempt are above the diagonal while for without suicide attempt are below the diagonal. *p ≤0.05; **p ≤0.01.

For the individuals with suicide attempt (**Table 2**, right triangle), ideation was highly positively correlated with depression (*r*_with_ = .64, *p* <.001) and moderately with hopelessness (*r*_with_ = .51, *p* <.001), and negatively with perceived personal strength subscale (*r*_with_ = -.49, *p* <.001). Depression was only significantly correlated with ideation, highly with hopelessness (*r*_with_ = .89, *p* <.001), and moderately negatively with perceived personal strength scale (*r*_with_ = -.60, *p* <.001). Hopelessness reveals a significantly correlation with ideation and depression, and a negative moderate correlation with perceived personal strength scale (*r*_with_ = -.65, *p* <.001).

**Figure 2:**
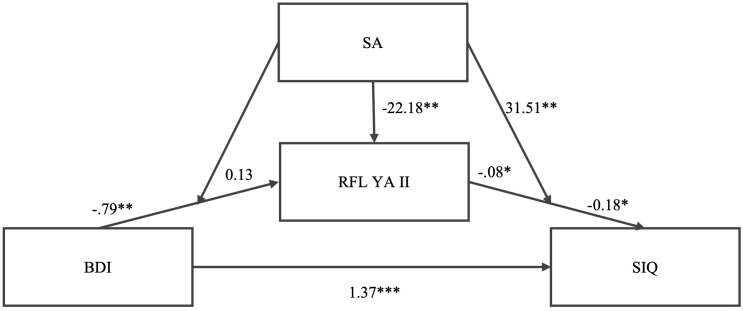
Tested model and no standardized coefficients.

Regarding the testing of the model (**Figure 2**), as shown in **Table 3**, higher levels of depressive symptomatology predict lower levels of reasons for living (*b* = -0.79, *SE* = 0.07, *p* = .001, *CI* = -.93, -.64). Previous suicide attempts did not moderate the association between depressive symptomatology and reasons for living, as the interaction was not significant (*b* = 0.13, *SE* = 0.24, *p* = .587, *CI* = -.34, -.59). Predictors explained 18.5% of reasons for living (*R* ^2^=.19; *F(3,84)* = 63.41, *p* <.001).

**Table 3:** Moderated mediation results for the link between depressive symptomatology and suicidal ideation. .

Antecedent	Consequent									
	Mediation Model M (RFL-Ya-II)					Dependent Model Y (SIQ)				
	*b*	*SE*	*t*	*p*	*CI*	*b*	*SE*	*t*	*p*	*CI*
X (BDI)	-.79	.074	-10.05	.001	(-.93; -.64)	1.37	.05	25.18	.000	(1.26; 1.48)
M (RFL-YA-II)	–	–	–	–	–	-.08	.03	-3.18	.015	(-.13; -.03)
W (TS)	-22.18	6.62	-3.50	.001	(-35.18; -9.18)	31.51	8.79	3.58	.000	(14.24; 48.78)
Int	.13	.24	.54	.590	(-.34;.59)	-.18	.09	-2.12	.040	(-.35; -.01)
Constant	132.61	1.05	126.75	.000	(130.56;134.66)	11.01	3.49	3.15	.020	(4.16; 17.88)
	R^2^=.19					R^2^=.55				
	F(3,84)=63.41, *p*<.001					F(4,84)=252.93, *p*<.001				

Int: for M – Int =X*W; for Y – Int=M*W.

Higher levels of reasons for living predict lower levels of suicidal ideation (*b* = -0.08, *SE* = 0.03, *p* = .015, *CI* = -.13, -.03). History of suicide attempts moderates the association between reasons for living and suicidal ideation (*b* = -0.18, *SE* = 0.09, *p* = .035, *CI* = -.35, -.01), as the interaction is significant.

The moderated mediation model showed that the indirect effect was only significant for reasons for living and the interaction between reasons for living and suicide attempts (path b), but not for depression and reasons for living (path a); that is, the moderator has mediation influence on the dependent variable, but not of the independent variable on the mediator.

The moderated mediation effect is supported by the significant indirect effect of depressive symptomatology on suicidal ideation through reasons for living only in the group of individuals without a history of suicide attempts. The moderated mediation is significant when 95% CI did not encompass zero as occurs in the no suicide attempt group. The predictors explain 55% of the variance in suicidal ideation.

## 4 Discussion and conclusion

Suicide is usually the culmination of a continuous process that includes other phenomena, such as self-injurious behaviors, suicidal ideation, or suicide attempts (3, 9, 13).

Within the study of suicidal behaviors, empirical studies include both risk and protective factors in understanding suicidal behavior, highlighting the role of the protective factors (35). This study aimed to assess the relationship between protective factors (i.e., reasons for living) and risk factors (i.e., depressive symptoms and hopelessness) for suicidal ideation and verify whether reasons for living mediate the relationship between risk factors and suicidal ideation in young adults with and without a history of suicide attempt.

The comparison between individuals with history of suicide attempt, our results showed that there are significative differences between them. The group with suicide attempt reveals predominance of suicidal ideation, depressive symptoms and feelings of hopelessness, and lower scores of the reasons for living. These results are accordingly to previous studies (3, 4, 8, 36, 37). The Christensen et al. (37) study, for example, also with young adults, found that individuals with a history of suicide attempts and suicidal ideation reported significantly lower reasons for living than individuals with no history of suicide attempts, even if they had a history of suicidal ideation. Only in the sub-scale Faith-Related Support of the Reasons for Living measure no significative differences were reported, but a moderate effect size was described. Although faith is frequently pointed as a protective factor, we did not find it in the present study. This could be due to the sample size or to the fact that the sample is composed of young adults, maybe with less consolidate beliefs.

The correlations’ analysis corroborates the previous research (3–5) since the group with suicide attempt showed positive associations with the risk factors (e.g., depression, hopelessness) and negative with the protective factors (e.g., reasons for living). Regarding the group without suicide attempt, the results reveal positive and moderate to strong relations with the protective factors, which could act as a subjacent cause to mitigate potential risks toward suicide behaviors (4, 16). Moreover, the tested model also showed that the reasons for living can be a negative mediator of the relation between the depressive symptoms and suicidal ideation, that is, it can act as a protective factor. Another relevant relation tested in the model was that a history of suicide attempt can be a moderator between Reasons for Living and suicidal ideation, intensifying this connection.

These results indicate that coping and survival beliefs and responsibility with family may be considered protective factors for high-risk people (38). Individuals with high levels of reasons to live may exhibit high levels of fear of suicide, fear of social disapproval, moral objections to suicide, coping and survival beliefs, and responsibilities to family. These people may therefore have negative attitudes toward suicide and positive attitudes toward themselves and life. Thus, even individuals who have higher levels of reasons for living may believe that they cannot escape a particular situation, yet they may choose more constructive ways, rather than suicide, as solutions for coping with aversive situations (23). It is therefore understood that reasons for living are a relevant domain that should be addressed earlier in interventions to prevent and cope with suicidal ideation and to reduce the risk of suicide (6, 13, 20, 23, 35, 39).

The results obtained in Bruns and Letcher’s (35) research support the hypothesis that higher levels of protective factors may be associated with lower levels of suicide risk. The study of Shi et al. (36) also highlights the role of reasons for living as protective factors, having simultaneously assessed depressive symptoms and hopelessness, among others, as risk factors for suicide. Thus, it is important that prevention of suicidal behaviors aim to promote the development or enhancement of protective factors and reduction or minimization of risk factors (40), such as at the level of clinical intervention.

Young adults are vulnerable to suicide, which seems to be strongly related to family conditions and the sociocultural context. This vulnerability is mainly the result of the interaction of multiple factors and, therefore, intervention strategies for these behaviors should consider transdisciplinary approaches (41) and considered the development of internal assets as resilience (42).

In the present study, all methodological procedures required in a research study were followed, however there are some limitations, mainly regarding the used samples. There was a big difference between the “clinical” and non-clinical sample size, and the representation of young adults with a history of suicide attempt may be underestimated in this study, although these population are not very easy to have access and to participate.

Despite the limitations, the evidence found in this study allows us to verify that reasons for living may play a preponderant role in suicidal ideation. An intervention focused on promoting these beliefs and developing strategies to face adversity and deal with life situations that may cause psychological pain and suffering may work as a protective measure for individuals with a history of suicide attempt, or who may be more susceptible to suicidal ideation. Thus, it is important that professionals who intervene with these mental health situations are aware of the protective factors and recognize the role of reasons for living in the prevention and clinical intervention of suicidal behaviors.

## Data Availability

The raw data supporting the conclusions of this article will be made available by the authors, without undue reservation.
